# Pathophysiological Consequences of At-Risk Alcohol Use; Implications for Comorbidity Risk in Persons Living With Human Immunodeficiency Virus

**DOI:** 10.3389/fphys.2021.758230

**Published:** 2022-01-18

**Authors:** Liz Simon, Scott Edwards, Patricia E. Molina

**Affiliations:** Department of Physiology and Comprehensive Alcohol-HIV/AIDS Research Center School of Medicine, Louisiana State University Health Sciences Center, New Orleans, LA, United States

**Keywords:** alcohol, HIV, cardiometabolic comorbidity, neuropathology, diet

## Abstract

At-risk alcohol use is a significant risk factor associated with multisystemic pathophysiological effects leading to multiorgan injury and contributing to 5.3% of all deaths worldwide. The alcohol-mediated cellular and molecular alterations are particularly salient in vulnerable populations, such as people living with HIV (PLWH), diminishing their physiological reserve, and accelerating the aging process. This review presents salient alcohol-associated mechanisms involved in exacerbation of cardiometabolic and neuropathological comorbidities and their implications in the context of HIV disease. The review integrates consideration of environmental factors, such as consumption of a Western diet and its interactions with alcohol-induced metabolic and neurocognitive dyshomeostasis. Major alcohol-mediated mechanisms that contribute to cardiometabolic comorbidity include impaired substrate utilization and storage, endothelial dysfunction, dysregulation of the renin-angiotensin-aldosterone system, and hypertension. Neuroinflammation and loss of neurotrophic support in vulnerable brain regions significantly contribute to alcohol-associated development of neurological deficits and alcohol use disorder risk. Collectively, evidence suggests that at-risk alcohol use exacerbates cardiometabolic and neurocognitive pathologies and accelerates biological aging leading to the development of geriatric comorbidities manifested as frailty in PLWH.

## Development of Alcohol Use Disorder

Globally, at-risk alcohol use is the seventh leading cause for morbidity and mortality accounting for 5.3% of all deaths, and the leading cause of disability-adjusted life-years (DALYs) among individuals 15–49 years of age (Burton and Sheron, 2018). Alcohol use disorder (AUD) is a chronic, relapsing condition often characterized by a parallel emergence of neurological deficits and negative emotional states (e.g., cognitive dysfunction, depression, pain) along with a significant escalation of alcohol use (Edwards and Koob, 2010). Consequently, the progression from recreational to at-risk drinking is thought to involve a motivational transition from positive reinforcement (i.e., drinking for pleasure) to negative reinforcement (i.e., drinking to avoid or mask the unpleasant symptoms of withdrawal). However, continued drinking at high levels over long periods of time not only continues to damage the brain and other organs, but also locks the individual into a motivational cycle of binge/intoxication, withdrawal, and craving/anticipation of renewed drinking during repeated abstinence (Koob, 2021). The primary driver of excessive alcohol drinking stems from a dysregulation of motivational behaviors regulated by the brain. Specific brain reinforcement centers (including the central amygdala and prefrontal cortex described below) have evolved to process adaptive interactions with natural rewards encountered in our environment (including the pursuit of food, sex, and social cooperation). However, the adaptive function of these brain regions and goal-directed behaviors can be compromised by multiple genetic and environmental factors, including the excessive use of alcohol.

Epidemiological data using Diagnostic and Statistical Manual of Psychiatric Disorders (DSM-V) criteria indicate that 13.9% of the United States of America (United States) population met criteria for AUD over the past year (Grant et al., 2015). AUD significantly decreases life expectancy and increases the risk of mortality from mental disorders (10-fold), and from cardiovascular diseases and cancers (two-fold) (Roerecke and Rehm, 2014). The increased risk of mortality associated with at-risk alcohol use results from alcohol-induced end organ injury spanning cardiopulmonary, gastrointestinal, immune, adipose, musculoskeletal, and nervous systems.

### Relevance of Alcohol Drinking Patterns

Drinking in moderation, is defined by the 2015–2020 Dietary Guidelines for Americans as consumption of up to one drink per day for women and up to two drinks per day for men. Low risk drinking as defined by the National Institute on Alcohol Abuse and Alcoholism (NIAAA) is no more than 3 drinks per day and no more than 7 drinks per week for women, and no more than 4 drinks per day and no more than 14 drinks per week for men. Drinking in moderation and low risk drinking are not associated with a significant increase in risk for alcohol-induced comorbidities. In contrast, binge drinking defined as drinking to elevate blood alcohol concentrations to 0.08% (80 mg/dl) or higher, generally resulting from drinking 4 or 5 drinks over a 2-h time frame is considered at-risk and categorized as extreme when the consumption is twofold or greater than the gender-specific thresholds (i.e., 10 or more standard drinks for men, and 8 or more for women) (Yeomans et al., 2003; Alcohol Research, Current Reviews, and Editorial Staff., 2018). Alcohol consumption is discouraged in certain groups of people including underage individuals (less than 21 years of age), women who are pregnant or trying to become pregnant, those who take medications that interact with alcohol, and those with existing medical conditions, including human immunodeficiency virus (HIV) as discussed in this review (Alcohol Research, Current Reviews, and Editorial Staff., 2018).

While the contribution of at-risk alcohol consumption to organ injury is well recognized, the definition of at-risk drinking is complicated by different patterns of consumption, the types of alcoholic beverages consumed across geographical regions (World Health Organization, 2021), and the inconsistent definition of a standard drink across countries, ranging from 8 g in Iceland to 20 g in Austria (Kalinowski and Humphreys, 2016). In addition, there appears to be wide variability for defining rates of low to moderate-risk drinking across nations, ranging from 98 to 140 g per week for women and 150–280 g per week for men (Kalinowski and Humphreys, 2016). Several factors are considered in the classification of alcohol drinking patterns, including the frequency and amount of alcohol intake, number of episodes of acute intoxication, and number of alcohol binges (Wetterling et al., 1999). According to the World Health Organization (WHO), prevalence of at-risk drinking is measured by heavy episodic drinking, defined as consuming 60 g of alcohol or more on at least one occasion in the past 30 days. In the United States, where a standard drink is 14 g, heavy episodic drinking is defined by consumption of 4.25 standard drinks. Worldwide, approximately 18% of the adult population report heavy episodic drinking (Poznyak, 2018).

## At-Risk Alcohol Use in Chronic Disease: Relevance to Persons Living With Human Immunodeficiency Virus

The Centers of Disease Control (CDC) estimates that over 50% of PLWH in the U.S. are 50 years of age or older (CDC, 2018). As life expectancy of PLWH continues to rise and approach that of the general population, the frequency of maladaptive behaviors increases as well. Maladaptive coping resulting from psychosocial stressors, including stigma associated with living with HIV, is associated with poorer immune status, increased viral load over time, faster disease progression, and higher rates of mortality (Cruess et al., 2003; Leserman, 2008). At-risk alcohol use is among the principal maladaptive coping behaviors in PLWH, and AUD frequently occurs in PLWH (Wardell et al., 2018).

AUD may exacerbate the risk for geriatric comorbidities including cardiometabolic syndrome (CMS) and neurological deficits (Kahl and Hillemacher, 2016; Sullivan and Pfefferbaum, 2019; Edwards et al., 2020) among PLWH. Data collected from an ongoing New Orleans Alcohol and HIV (NOAH) longitudinal clinical study (Ferguson et al., 2020) show that lifetime alcohol exposure positively associates with Phenotypic Frailty Index and a 52-item deficit index after adjustment for subject demographics, HIV-related covariates, smoking, and history of other substance use (Maffei et al., 2020). Premature decline in functional status has been reported in PLWH (Piggott et al., 2016). Data show that the prevalence of a frailty-related phenotype (unintentional weight loss, exhaustion, weakness, slow walking speed, low physical activity) in 55-year-old HIV-infected men is comparable to that of HIV-uninfected men > 65 years old, strongly supporting accelerated biological aging in PLWH and its likelihood of associated enhanced risk for comorbidities (Desquilbet et al., 2007).

The principal mechanisms underlying alcohol addiction, alcohol-induced organ injury, and alcohol-related liver injury have been extensively reviewed (Molina et al., 2014b; Massey et al., 2015; Aberg et al., 2020; Arab et al., 2020; Maddur and Shah, 2020; Rasineni et al., 2020; Koob, 2021; Osna et al., 2021). Here we focus on salient mechanisms of alcohol-induced risk and exacerbation of CMS and neuropathological comorbidities and discuss their implications in the context of HIV infection and their role in exacerbating or accelerating development of associated comorbidities in PLWH.

## Mechanisms of Alcohol-Induced End Organ Injury

**FIGURE 1:**
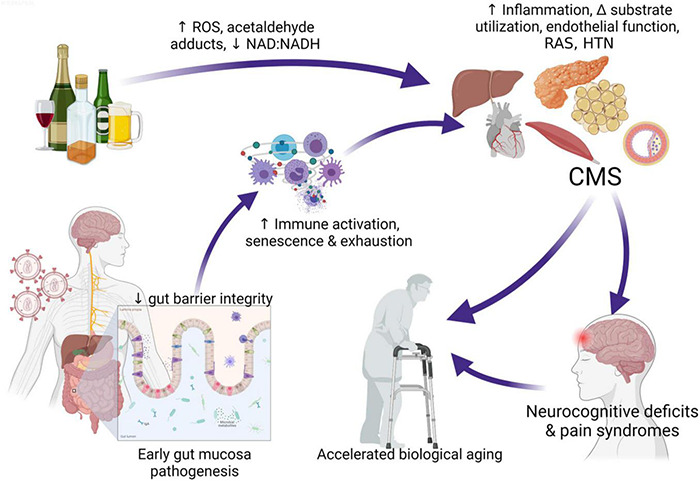
Interaction of at-risk alcohol use and HIV infection. Chronic stressors including unhealthy alcohol consumption and HIV infection affect several biological processes and synergize with gut immunopathological effects of alcohol, decreasing gut mucosal barrier integrity, promoting dysbiosis, and gut bacteria and toxin leak. Alcohol metabolism generates toxic by-products that, in turn, can promote tissue and cell injury. Pathophysiological processes frequently linked to alcohol metabolism including oxidative stress, mitochondrial injury, altered growth factor signaling, nutritional deficits, and epigenetic modifications. These synergize with systemic immune activation, inflammation, and cell senescence and exhaustion driven by gut leak, enhancing tissue injury and dysregulation of homeostatic mechanisms increasing risk for cardiometabolic syndrome (CMS). The cluster of target organ dyshomeostasis associated with CMS is associated with increased risk for neurocognitive deficits and pain syndromes that can exacerbate or accelerate biological aging. ROS, reactive oxygen species; RAS, renin angiotensin system; HTN, hypertension; CMS, cardiometabolic syndrome. Created with Biorender.com.

Alcohol-induced tissue injury leading to increased risk of comorbidities result from a combination of pathophysiological processes frequently linked to alcohol metabolism including oxidative stress, mitochondrial injury, altered growth factor signaling, nutritional deficits, and epigenetic modifications (Molina et al., 2014b). Alcohol metabolism can occur in virtually all tissues but occurs predominantly in the liver and generates toxic by-products that, in turn, can promote tissue and cell injury (Souza-Smith et al., 2016). Following first-pass metabolism of a small fraction of ingested alcohol in the stomach, most (92–95%) alcohol consumed is degraded by alcohol dehydrogenase (ADH) and aldehyde dehydrogenase (ALDH), forming acetaldehyde and acetate. Alcohol breakdown to acetaldehyde by ADH is associated by nicotinamide adenine dinucleotide (NAD^+^) reduction to NADH and a resulting decreased NAD^+^: NADH ratio that decreases availability of mitochondrial glutathione (mGSH), and cellular antioxidant reserve. The highly reactive product of ADH; acetaldehyde, can react with proteins and cell membranes producing acetaldehyde–protein adducts that also contribute to tissue injury. Acetaldehyde metabolism to acetate by ALDH2 in the mitochondria, further decreases cellular NAD^+^: NADH ratio. In addition to ADH conversion of alcohol to acetaldehyde; alcohol is also metabolized by cytochrome P450, producing reactive oxygen species (ROS). Thus, alcohol and its metabolites, the generation of ROS, and formation of acetaldehyde adducts contribute to cell and tissue injury (Cederbaum, 2012). Oxidative stress and mitochondrial dysfunction are both implicated in HIV disease pathogenesis (Aukrust et al., 2003; Schank et al., 2021) suggesting that in combination, at-risk heavy alcohol use and HIV are likely to impose a greater oxidative burden on tissues. Chronic oxidative stress affects several biological processes and synergize with gut immunopathological effects of alcohol (Gu et al., 2021; Shukla et al., 2021) to decrease gut mucosal barrier integrity and promote dysbiosis (Maffei et al., 2021). The resulting gut leak promotes systemic immune activation, inflammation, and cell senescence, enhancing tissue injury and dysregulation of homeostatic mechanisms exacerbating the risk for comorbidities in PLWH (Katz et al., 2015; Maffei et al., 2020; Figure 1).

### Interactions of At-Risk Alcohol Use and Human Immunodeficiency Virus, Risk for Cardiometabolic Syndrome

Cardiometabolic syndrome is a combination of alterations including glucose intolerance, dyslipidemia, hypertriglyceridemia, central adiposity, and hypertension, that is now recognized by the WHO as a disease entity (Saljoughian, 2016). CMS increases mortality risk due to coronary heart disease, myocardial infarction, stroke, and type 2 diabetes (Castro et al., 2003) and is one of the geriatric comorbidities prevalent in PLWH (Woldu et al., 2020). Lifestyle behaviors including at-risk alcohol use, consumption of diets rich in fat and sugar, low physical activity, and behavioral and psychosocial stressors are associated with increased risk for CMS and type 2 diabetes. Alcohol use impacts risk for CMS following a U or J shape curve (Alkerwi et al., 2009; Baliunas et al., 2009; Koloverou et al., 2015; Lai et al., 2019). However, this is not generalizable across geographical locations or ethnic groups. Moderate alcohol consumption is shown to be protective in studies from the United States (Kerr and Ye, 2010) or Sweden (Rasouli et al., 2013), but not in studies conducted in Japan (Teratani et al., 2012). Data relating alcohol to all-cause and cardiovascular (CV) morbidity and mortality from more than 600,000 current drinkers without a history of CV disease, indicated a much lower threshold for amount of alcohol consumed than currently accepted (Wood et al., 2018). In a prospective, longitudinal study, male sex, being physically active, and in good health status were independently associated with light to moderate drinking. Though there was an apparent protective effect of light to moderate drinking on mortality after adjusting for age, sex, risk factors, and cardiovascular events, the relationship was no longer significant when adjusting for physical activity and perceived health status (Muscari et al., 2015). These results suggest that the protective effect of moderate alcohol use is most prominent among individuals with a healthier lifestyle (Koloverou et al., 2015). Moreover, compelling evidence suggest that risk for CMS increases with increasing alcohol consumption (Vieira et al., 2016). Alcohol-using PLWH have higher odds of displaying lipodystrophy (Cheng et al., 2009; Molina et al., 2014a) and altered adipokine profiles including that of leptin (Srdic et al., 2017) and adiponectin (Lee and Kwak, 2014; Langkilde et al., 2018). Alterations in these adipokines have been linked to metabolic dysregulation in obese subjects (Harwood, 2012; Lee and Kwak, 2014), in PLWH on ART (Jan et al., 2004; Lagathu et al., 2005), and to impaired insulin signaling (Jan et al., 2004; Stankov and Behrens, 2010). CMS is a comorbidity commonly associated with cognitive deficits in PLWH (Pope et al., 2020; Pasipanodya et al., 2021), and cardiometabolic health is linked to improved cognitive function in aging PLWH (Saloner et al., 2019).

Diet quality is influenced by the pattern of alcohol use, being the poorest among subjects who consumed the highest quantity of alcohol (Breslow et al., 2006), among women who were current drinkers, and among both men and women as the amount of alcohol consumption increased (Breslow et al., 2010). The interaction of unhealthy alcohol use with an unhealthy diet is likely to further enhance risk for comorbidities. Diet quality among PLWH (Muhammad et al., 2019; Weiss et al., 2019) is significantly lower than recommended. In a large single-site study among PLWH in the US, dietary recall demonstrated that the average carbohydrate intake was twice the recommended amount and protein consumption was a third of recommendations and was also associated with a trend for increased fat intake (Webel et al., 2017). This dietary pattern, referred to as a Western Diet has been linked to immune and metabolic dysfunction (Childs et al., 2019). PLWH have a lower healthy eating index (HEI) score compared to HIV- subjects, with decreased scores for consumption of seafood and plant proteins and beneficial fatty acids, along with increased consumption of foods with non-nutritional calories including alcohol (Weiss et al., 2019). The HEI assesses dietary patterns and their degree of adherence to the Dietary Guidelines for Americans (Guenther et al., 2008), and scores ≤ 50 are inversely correlated with reduced risk of all-cause mortality in the general population (Sotos-Prieto et al., 2017a,b). Increased insulin, cholesterol, and triglycerides in PLWH are associated with increased total dietary energy intake with high total and saturated fat (Batterham et al., 2000). Chronic binge alcohol in simian immunodeficiency virus (SIV) infected macaques decreases total caloric intake and alters nutrient selection, decreasing nitrogen intake and balance as the disease progresses (Molina et al., 2006). The self-imposed carbohydrate rich, low nitrogen diet likely contributes to decreased muscle mass observed in end stage disease. Changes in dietary intake can influence micronutrient availability and in addition modulate circulating and tissue growth factors (Childs et al., 2019). Hence, direct, and indirect alcohol-mediated effects as well as diet quality and composition can significantly contribute to risk for CMS in PLWH.

## Mechanisms Implicated in Alcohol-Human Immunodeficiency Virus Interactions Increasing Risk and Pathogenesis of Cardiometabolic Syndrome

**FIGURE 2:**
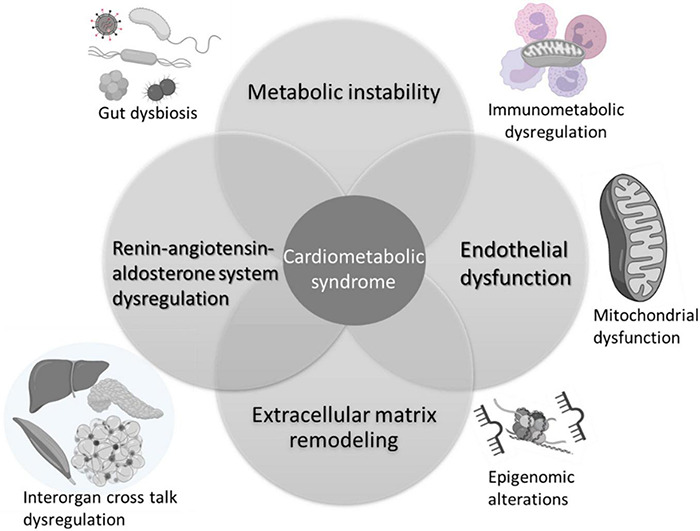
Mechanisms implicated in alcohol interactions with risk and pathogenesis of cardiometabolic syndrome. Alcohol associated metabolic instability, alterations in endothelial function, extracellular remodeling, and dysregulation of the renin-angiotensin aldosterone system are salient mechanisms that contribute to increased risk for CMS. Alcohol alters the gut microbiome, impacting on gut mucosal immunity and barrier function. The chronic immune activation associated with gut leak results in immune activation, exhaustion, and senescence, underlying immunometabolic dysregulation. Across tissues, alcohol produces alterations in cellular energy metabolism disrupting mitochondrial function and homeostatic responses. Several of these mechanisms are regulated by epigenomic alterations reflected by changes in microRNA profiles, histone methyltransferases and deacetylase expression, and activity. Alcohol-induced multi-organ alterations may result from inter-organ cross talk. Alcohol-induced tissue injury in one organ (i.e., adipose tissue) can result in release of mediators (i.e., adipokines, cytokines, microvesicles, etc.) that target distant organs amplifying alcohol’s deleterious effects. Created with Biorender.com.

The pathophysiology of CMS is multifactorial, involving multiple organs and several independent and interdependent pathways. Several mechanisms impacted by at-risk alcohol use can potentially increase the risk and/or exacerbate CMS in PLWH (Figure 2). The most salient ones are discussed below.

### Interference With Energy Substrate Utilization and Storage

**FIGURE 3:**
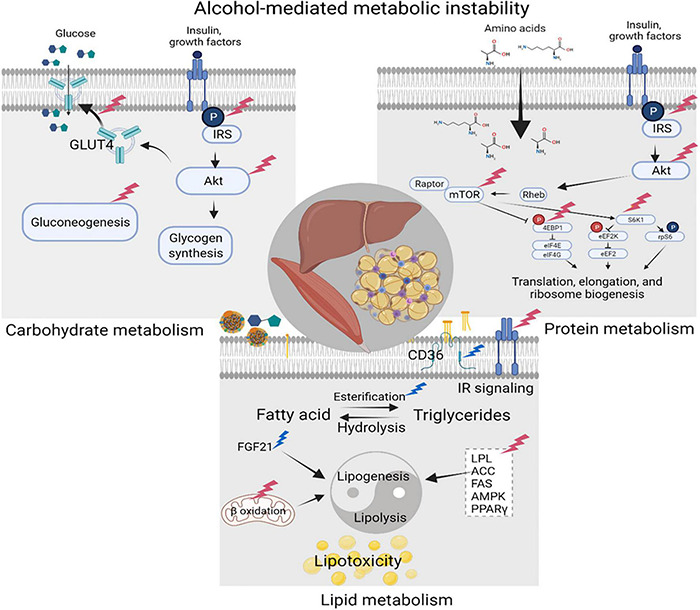
Alcohol-mediated metabolic instability. Alcohol produces carbohydrate, lipid, and protein metabolic dysregulation directly, and indirectly through the generation of its metabolites. Principal target organs include the liver, adipose tissue, and skeletal muscle (SKM). Alcohol-associated alterations in synthesis and breakdown of carbohydrates, lipids, and proteins, result in metabolic instability and increase the risk for cardiometabolic syndrome (CMS). Created with Biorender.com.

Alcohol-induced carbohydrate, lipid, and protein metabolic dysregulation is due to either direct effects of alcohol and its metabolites on liver, adipose tissue, and skeletal muscle (SKM), or due to indirect effects resulting from immune dysregulation (Figure 3). Alcohol consumption significantly decreases gluconeogenesis partly due to impaired hepatic utilization of lactic acid, glycerol, and alanine as gluconeogenic substrates (Steiner et al., 2015a) and a reduction in glycolytic and gluconeogenic hepatic enzyme activity (Baranyai and Blum, 1989; Mokuda et al., 2004). Alcohol impairs insulin signaling including decreased phosphorylation of the insulin receptor, insulin receptor substrate (IRS)-1, and AKT, and decreases GLUT4 membrane translocation in peripheral insulin sensitive tissues (Steiner et al., 2015a). Reports in the literature indicate that chronic alcohol feeding (Kang et al., 2007b) as well as ART (Boccara, 2008) result in the development of insulin resistance, one of the main components of CMS. The mechanisms underlying insulin resistance and dysglycemia; manifested as an increased frequency of glucose intolerance or frank diabetes mellitus in PLWH (Willig and Overton, 2016; Natsag et al., 2017; Noumegni et al., 2017), remain poorly understood. Chronic subclinical inflammation has been proposed as a likely factor contributing to metabolic dyshomeostasis seen in ART-treated PLWH (Longenecker et al., 2013; Beires et al., 2018).

SKM is the major insulin-sensitive tissue in the body and is a major player in maintaining glucose homeostasis. Thus, alcohol-induced alterations in SKM homeostasis can potentially have a significant impact on metabolic dysregulation. The alcohol-mediated decrease in SKM protein synthesis (LeCapitaine et al., 2011; Steiner and Lang, 2019) together with alterations in the mammalian target of rapamycin (mTOR) signaling pathway (Hong-Brown et al., 2012) result in alcoholic myopathy; which together with the alcohol-associated alterations in synthesis and breakdown of carbohydrates and lipids, contribute to metabolic instability and increased risk for CMS (Figure 3). These alcohol-mediated alterations in SKM and adipose mass, and carbohydrate storage, can be reflected in anthropometric changes as well as in alterations of circulating levels of glucose, triglycerides, and cholesterol, as briefly discussed in the next section.

### Alterations in Body Composition

Central or abdominal obesity is associated with increased insulin resistance (Klaus et al., 2009; Govers, 2015; Jankie and Pinto Pereira, 2021). Heavy (20 to less than 60 g/day) and very heavy (60 g/day) alcohol use increases waist circumference and abdominal fat accumulation (Churilla et al., 2014; Hirakawa et al., 2015). Though the exact mechanisms are unclear, proposed alcohol-associated mechanisms include decreased leptin and glucagon like peptide -1, alterations in sex steroid hormones, loss of lean body mass, and increased lipolysis and fatty acid flux (Riserus and Ingelsson, 2007; Sumi et al., 2019). In the post-ART era, obesity is emerging as a critical problem in PLWH (Godfrey et al., 2019), with increased body mass index (BMI) associated with increased risk of CV disease and diabetes mellitus among PLWH (Achhra et al., 2018).

### Alterations in Glycemic Control

Reports on the effects of alcohol on glycemic control are mixed. Low to moderate alcohol use inhibits hepatic glycogenolysis and gluconeogenesis, decreases fasting insulin and HbA1c, and increases insulin sensitivity (Hong et al., 2009; Koloverou et al., 2015; Schrieks et al., 2015; Vieira et al., 2016). Clinical studies show that alcohol decreases circulating basal insulin secretion (Bonnet et al., 2012) and decreases circulating insulin and c-peptide response to glucose (Patto et al., 1993). Preclinical studies show that chronic binge alcohol significantly impairs endocrine pancreatic response to a glucose load in SIV-infected macaques (Ford et al., 2016; Simon et al., 2021). Similarly, rodents on a chronic alcohol diet have decreased circulating insulin levels (Rasineni et al., 2019), and decreased pancreatic expression of glucokinase, glucose transporter-2 (Kim et al., 2010), and gamma-aminobutyric acid (GABA) receptors (Wang et al., 2014). These alcohol-induced alterations can potentially contribute to decreased insulin release following a glucose load. This is aligned with studies showing that *in vitro* alcohol exposure decreases glucose-stimulated insulin secretion (GSIS) from human (Dragan et al., 2017) and rodent (Wang et al., 2014; Rasineni et al., 2019) pancreatic islets and increases β-cell apoptosis (Dembele et al., 2009; Kim et al., 2010). These findings support a role for alcohol-mediated impairment of pancreatic endocrine function, particularly of the integrity of β-cell response to glucose as an underlying mechanism of glucose dyshomeostasis. In addition, preclinical studies show that adiponectin, an insulin sensitizing adipokine with anti-inflammatory properties is decreased with at-risk alcohol use (Ford et al., 2016; Steiner and Lang, 2017). A potential factor in alcohol-mediated decrease in adiponectin levels may be increased resistin levels in both clinical studies and animal models of alcohol use. Resistin suppresses adiponectin secretion and stimulates lipolysis, releasing fatty acids and glycerol into circulation (Chen et al., 2014). However, results from clinical studies are inconclusive, with both low to moderate and at-risk alcohol use increasing adiponectin (Steiner and Lang, 2017; Nova et al., 2019). Overall, alcohol-associated alterations in adipocytokine profile may contribute to impaired glucose homeostasis. Moreover, alcohol-mediated impairment of glycolytic pathways is associated with increased formation of advanced glycation end products (AGEs), which together with endothelial dysfunction, inflammatory responses, and oxidative stress discussed below contribute to the development of hypertension and atherosclerosis, important components of CMS (Vasdev et al., 2006). Recent data show that in PLWH at-risk alcohol use increased the likelihood of meeting the clinical criteria for prediabetes/diabetes (Primeaux et al., 2021). Moreover, HOMA-β cell function negatively associated with AUDIT-C, phosphatidyl ethanol (PEth), and Timeline Followback (TLFB), suggesting that alcohol use is associated with impaired endocrine pancreatic function (Simon et al., 2020). Others have also reported a high incidence of diabetes in well controlled PLWH (Bratt et al., 2021), associated with increased oxidative stress (Bastard et al., 2019).

### Dysregulation of Lipid Homeostasis

Chronic alcohol use results in a dose-response positive relationship with high density lipoprotein-cholesterol (HDLc) and negative relationship with low density lipoprotein (LDL) (Shuval et al., 2012). The alcohol-mediated biological mechanisms contributing to lowering of cholesterol levels include increased transport of lipoproteins and lipoprotein lipase activity (Shuval et al., 2012), and increased eNOS activity that helps transport of HDL-C (Hirakawa et al., 2015; Wakabayashi, 2019). At-risk alcohol use increases triglyceride levels especially among women (Hirakawa et al., 2015; Vieira et al., 2016) and moderate alcohol is associated with lower triglyceride levels (Kovar and Zemankova, 2015). Alcohol-mediated mechanisms that contribute to increased triglycerides include adipose tissue lipolysis, and hepatic synthesis of large very low density lipoprotein (VLDL) particles through the increased expression of microsomal triglyceride transfer protein (Klop et al., 2013). The interactions of alcohol with high fat diet further exacerbate hypertriglyceridemia by regulating hypothalamic peptides that increase appetite and energy intake (Barson et al., 2010).

Alcohol directly and indirectly alters the balance of lipogenesis and lipolysis and thus dysregulates lipid metabolism (Figure 3). Loss in adipose tissue mass resulting from chronic alcohol consumption is partly due to an increase in triglyceride turnover without significant alteration in triglyceride synthesis (Kang et al., 2007a; Zhong et al., 2012). Alcohol-induced activation of lipolysis and release of free fatty acids (FFAs) that are taken up by the liver has been proposed as a central mechanism of alcohol-induced steatohepatitis. Overall, alcohol alters hepatic lipid flux by increasing free fatty acid uptake from the diet and from adipose tissue lipolysis; stimulating lipogenesis from glycolytic end products; and dysregulating β- oxidation. Moreover, alcohol upregulates expression of fatty acid transporters particularly CD36/FAT promoting fatty acid uptake, and esterification of free fatty acids into triglycerides (You and Arteel, 2019). Alcohol dysregulates lipolysis through inhibitory effects on insulin action (Yki-Jarvinen et al., 1988), but not through enhanced adrenergic effects (Lang et al., 2014). In addition, alcohol increases plasma and adipose tissue expression of fibroblast growth factor 21 (FGF21), a known stimulus for lipolysis (Zhao et al., 2015). In contrast, alcohol decreases expression and activation of several lipogenic enzymes (Steiner and Lang, 2017). Thus, alcohol-associated dysregulation of lipid homeostasis results from an imbalance between lipolysis and lipogenesis. Dyslipidemia is highly prevalent among PLWH (Grunfeld et al., 1992), and this has been attributed to the infection itself, and some types of ART drugs. Early following HIV seroconversion, total, HDL, LDL cholesterol levels decrease. Following the initiation of ART, the profile shifts to increased total and LDL cholesterol to pre-infection levels, with persistent low HDL levels (Riddler et al., 2003). Alcohol-using PLWH have higher odds of presenting with lipodystrophy (Cheng et al., 2009). CVD risk factors, including dyslipidemia and hypertension, are determinants of reduced life expectancy in PLWH (Freiberg and Kraemer, 2010).

### Dysregulation of Blood Pressure

In addition to alcohol-induced metabolic dysregulation or instability, alcohol-associated risk for hypertension is also an important contributor to CMS (Saljoughian, 2016). Some studies report higher prevalence of hypertension in PLWH than in uninfected individuals, with a higher frequency in individuals 50 years of age or older (Xu et al., 2017; Fahme et al., 2018). Furthermore, hypertension in PLWH is associated with heavy alcohol use (Ikeda et al., 2013). Multiple mechanisms affected by alcohol and HIV infection can contribute to blood pressure dysregulation. Alcohol-induced dysregulation of vasoconstrictor levels, vasoreactivity, and endothelial integrity are all possible mechanisms contributing to altered blood pressure regulation. Alcohol at low doses produces transient vasodilation. In contrast, at higher doses and with binge drinking, alcohol leads to vasoconstriction (Rekik et al., 2002; Kloner and Rezkalla, 2007) and chronic heavy alcohol use increases the risk of hypertension (Kahl and Hillemacher, 2016; Vieira et al., 2016). Several mechanisms contribute to alcohol-associated hypertension including genetic predisposition for an *ALDH2* polymorphism (Hu et al., 2014), activation of renin angiotensin aldosterone system (RAAS), central adrenergic activity, increased sympathetic flow, vascular smooth muscle tone, shifts in baroreceptor reflex sensitivity, and decreased nitric oxide (NO) release (Husain et al., 2014; Kahl and Hillemacher, 2016). In addition, alcohol increases both cytosolic free calcium (Ca^2+^) and cellular Ca^2+^ uptake by direct upregulation of voltage-gated Ca^2+^ channels, inhibits Ca^2+^-adenosine triphosphatase, and sodium-potassium ion pumps, thus increasing sensitivity to endogenous vasoconstrictors and exacerbating hypertension (Altura and Altura, 1982; Husain et al., 2014). The magnitude of increase in blood pressure with heavy alcohol use averages about 5–10 mm Hg with greater increases in systolic than diastolic pressure (Clark, 1985). The association between alcohol use and hypertension is stronger among older individuals (Burke et al., 1992; Riserus and Ingelsson, 2007), which may be due to enhanced sensitivity to sympathoadrenal activation (Husain et al., 2014). Thus, alcohol-associated risk for hypertension results from alterations in endothelial function and in the levels and activity of endogenous mediators of vasoreactivity as discussed in the next section.

### Endothelial Dysfunction

**FIGURE 4:**
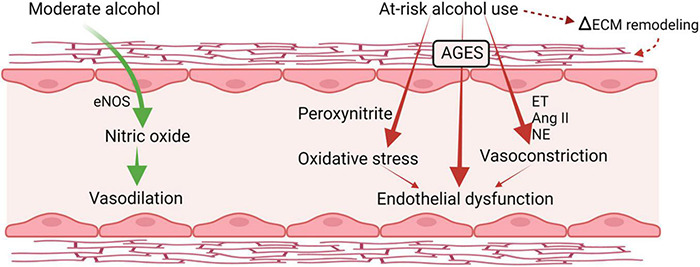
Alcohol-induced biphasic effects on endothelial function. Acutely, moderate doses of alcohol increase production of nitric oxide and result in transient vasodilation. Chronic, heavy alcohol consumption produces oxidative stress and activates vasoconstrictor pathways including endothelins (ET) and angiotensin II formation (Ang II) that lead to endothelial dysfunction and altered response to vasoactive mediators. ET, endothelin; Ang II, angiotensin II; NE, norepinephrine; eNOS, endothelial nitric oxide synthase; AGES, advance glycation end products; ECM, extracellular matrix. Created with Biorender.com.

Several alterations in vascular integrity and responsiveness may contribute to increased risk for hypertension and vascular instability. The vascular endothelium integrates humoral and hemodynamic signals modulating vasomotor tone and adjusting blood flow to the local tissue needs. Alcohol exerts biphasic effects on endothelial function (Puddey et al., 2001; Figure 4). Acute alcohol consumption produces immediate and transient vasodilation (Bau et al., 2005; Oda et al., 2020) followed by increased blood pressure later after alcohol consumption resulting in a biphasic effect of alcohol on blood pressure. The acute alcohol-induced vasodilation is mainly attributed to the increased expression of endothelial nitric oxide synthase (eNOS) and NO (Venkov et al., 1999). This mechanism has been proposed in cardioprotective benefits of moderate alcohol consumption (Abou-Agag et al., 2005; Toda and Ayajiki, 2010). Chronic and at-risk alcohol use is associated with hyperactivation of the hypothalamo-pituitary-adrenal (HPA) axis and increased glucocorticoid release. Excess glucocorticoid action can also contribute to endothelial dysfunction and accelerate the atherogenic process (Bau et al., 2005). Overall, at-risk alcohol use increases the risk of coronary artery disease, and hemorrhagic and ischemic stroke. Endothelial dysfunction is a predictor of CV disease and precedes atheromatous plaque formation (Schachinger and Zeiher, 2000). In addition, cytotoxicity resulting from at-risk alcohol use through generation of peroxynitrite, resulting from reaction of NO with superoxide; a product of alcohol-induced oxidative stress (Pacher et al., 2007), may also contribute to endothelial dysfunction. PLWH have significant impairment in endothelial function reflected in significantly lower flow-mediated vasodilation compared to uninfected controls, and present with elevated levels of markers of endothelial dysfunction (Rethy et al., 2020). Though evidence supports a deleterious effect of HIV infection on endothelial function, definitive studies remain limited and inconclusive.

### Dysregulation of Vasoconstrictor Levels and Vasoreactivity

At-risk alcohol use may alter the balance of endogenous vasoconstrictor levels including angiotensin II, endothelins, and norepinephrine (Tsuji et al., 1992), contributing to the increased risk for hypertension. Studies demonstrate that alcohol consumption increases overall activity of RAAS (Jing et al., 2008; Yarlioglues et al., 2019). Plasma renin activity is increased in individuals with heavy alcohol consumption (Husain et al., 2014) and angiotensin converting enzyme (ACE) activity in serum of hospitalized individuals with history of chronic alcohol consumption is higher than that of healthy controls (Okuno et al., 1986). Similarly, some evidence supports a role for increased RAAS activation in development of hypertension and metabolic syndrome in PLWH (Srinivasa et al., 2015; Masenga et al., 2020). The importance of increased activation of the Ang II type 1 receptor (AT1) to the development of alcoholic cardiomyopathy has been shown in preclinical studies (Cheng et al., 2006). Furthermore, activation of the AT1 receptor by chronic alcohol activates NADPH oxidase producing endothelial injury (Warnholtz et al., 1999; Gonzaga et al., 2018; Assis et al., 2020). Alcohol increases levels of renin, aldosterone, and angiotensin II (Jing et al., 2008), circulating renin and ACE activity, and left ventricular myocyte AT1 receptor expression in individuals with at-risk alcohol use (Husain et al., 2014). In addition, at-risk alcohol use results in increased plasma vasopressin levels indicating increased sympathetic stimulation (Chan and Sutter, 1983). Together with alterations in endothelial function, the alcohol-induced dysregulation of circulating levels of endogenous vasoactive mediators and their respective receptors promotes alterations in blood pressure regulation, increasing the risk for hypertension.

#### Alcohol-Human Immunodeficiency Virus Interactions Increase Risk for Neurological Impairment in Persons Living With Human Immunodeficiency Virus

CMS has been linked to neurocognitive impairment in the general population (Yaffe et al., 2004; Panza et al., 2010), particularly in the presence of inflammation. More recently, this link has been examined in the context of HIV infection, demonstrating an additive effect of HIV and CMS on neurocognitive dysfunction, including apathy and decreased executive function (Pope et al., 2020), and impairments in the neurocognitive domains of learning and fine motor skills (Yu et al., 2019). The neurological consequences of chronic HIV infection span both the peripheral and central nervous systems, and despite the success of ART to control viral loads, HIV infection and treatment continue to promote neurological and psychiatric co-morbidities that worsen disease outcomes and overall quality of life. Neurocognitive disorders are highly prevalent in PLWH, and HIV-associated neurocognitive disorder (HAND) and co-occurring AUD can exacerbate these deficits (Fama et al., 2016). HAND can include deficits in attention, memory, and executive function. ARTs have successfully extended life expectancy of PLWH but are not fully protective against the neurocognitive symptoms of HAND and therefore greater cognitive impairment may surface with aging (Alakkas et al., 2019).

The cognitive and behavioral deficits associated with excessive drinking are commonly attributed to long-lasting neuroadaptations and functional changes to neuronal circuitry, as both former and current AUD subjects demonstrate cognitive impairments including deficits in working memory (Kopera et al., 2012), executive functioning (Green et al., 2010), and impulsivity (Li et al., 2009), which all contribute to maladaptive decision-making, including relapse to alcohol seeking during attempted abstinence periods. Interestingly, such deficits partially but incompletely overlap between men and women (Fama et al., 2020). From a neuroanatomical perspective, these chronic alcohol induced-cognitive impairments are associated with selective brain damage in vulnerable areas such as the prefrontal cortex (Pfefferbaum et al., 1998; Le Berre et al., 2014) and hippocampus (Mira et al., 2019). Heavy alcohol use may also predispose individuals to cognitive disorders such as Alzheimer’s disease (AD), as frequent drinkers display higher levels of AD-related biomarkers in their cerebrospinal fluid (Wang et al., 2021). In addition, a rapidly emerging area of interest is how sleep disturbances impact neurological function and may interact with cognitive deficits to promote AUD (Laniepce et al., 2021).

Neuroinflammation is hypothesized to be a primary mechanism of HAND-associated cognitive impairment (Garvey et al., 2014). However, it is becoming increasingly recognized that neuroinflammation alone cannot explain the neurological consequences of HIV (Gelman, 2015). In a murine model of HIVE, neuropathology persists despite suppressed neuroinflammation and CNS viral replication. Preclinical studies suggest that disruptions in growth factor expression and signaling likely contributes to HIV neuropathology and cognitive impairments (Maxi et al., 2016). Chronic binge alcohol unmasks neurocognitive deficits in SIV-infected, non-ART treated macaques (Winsauer et al., 2002). Furthermore, disruptions in growth factor expression and signaling as potential mechanisms contributing to cognitive impairments have been reported. Brain-derived neurotropic factor (BDNF) is a neuroprotective factor regulating synaptic plasticity and cognitive function. Reduced BDNF signaling through TrkB, including ERK and Akt pathways, have been shown to decrease neuronal plasticity and are linked to HIV-associated neurocognitive decline (Michael et al., 2020). *In vitro* studies have indicated that the HIV peptide *Tat* induces downregulation of *BDNF* and other CREB-regulated genes. Alcohol consumption can further reduce BDNF levels and impair BDNF signaling through TrkB receptors (John MacLennan et al., 1995). Interestingly, though ART attenuates expression of microglial markers of neuroinflammation in the frontal cortex and monocyte/macrophage markers of neuroinflammation in the basal ganglia, it did not ameliorate the alcohol-associated inhibition of growth factor signaling in the frontal cortex of SIV-infected macaques. These findings suggest that while ART may be effective in reducing neuroinflammation associated with infection and alcohol, it is not sufficient to attenuate the deficits in BDNF signaling and may explain the persistence of HAND despite widespread ART use (Maxi et al., 2019). These results support the need for continued research into strategies to prevent HIV-associated neurocognitive decline, including the potential to target neuroinflammation and growth factor signaling as new therapies are developed.

### Neuropathological Comorbidities Associated With At-Risk Alcohol Use and Human Immunodeficiency Virus

In addition to the direct and indirect alcohol-mediated peripheral tissue injury implicated in pathophysiology of CMS, at-risk alcohol use also impacts both peripheral and central nervous system physiology. This is perhaps best represented by alcohol’s ability to regulate the conscious experience of pain through its interactions with both ascending and descending nociceptive circuitry (Egli et al., 2012). Importantly, chronic pain affects approximately 20% of adults worldwide and approximately 100 million Americans (Nahin, 2015), a number that will likely increase over the next few decades given an aging U.S. population. Chronic or neuropathic pain is prevalent in approximately 57% of PLWH, significantly impairing quality of life (Ellis et al., 2010). PLWH with HIV-related pain also have higher rates of depression, anxiety, and insomnia, compared to those without neuropathy symptoms (Phillips et al., 2014), indicating that neurological pain symptoms may ultimately transform into CNS pathophysiology including cognitive dysfunction and psychiatric illness. Preclinical studies show significant heightened pain sensitivity in response to systemic injection of HIV viral particles (Guindon et al., 2019; Bagdas et al., 2020). SIV infection in pigtail macaques induces inflammatory cell infiltration in the spinal cord, a frequent finding in chronic pain conditions (Mangus et al., 2015).

Evidence also suggests that diet and metabolic factors influence the development and chronicity of pain symptoms. Diets containing high amounts of omega-6 fatty acids (common in Western diets) exacerbate nociceptive sensitivity in preclinical animal models of neuropathic and inflammatory pain (Boyd et al., 2021). Importantly, Western diets appear to prolong recovery from a pain-related injury in animals, although recovery was accelerated by switching animals to an anti-inflammatory diet (Totsch et al., 2018). Similar dietary interventions have also demonstrated efficacy in reducing pain symptoms in humans (Lattanzio and Imbesi, 2018). A review of global studies also highlighted links between affective disorders, metabolic syndrome, and chronic pain (Bica et al., 2017), strongly suggesting that negative affect serves as a moderator of these relationships. Convergent evidence suggests a strong underlying influence of chronic inflammation in these integrated disease processes, providing a mechanistic basis for how HIV and alcohol use promotes and sustains these conditions.

### At-Risk Alcohol Use, Chronic Pain, and Pain-Related Negative Affect

While the analgesic effects of alcohol have been known for some time (Thompson et al., 2017; Cucinello-Ragland and Edwards, 2021), excessive alcohol exposure damages elements of the peripheral nervous system producing a characteristic small fiber painful neuropathy (Maiya and Messing, 2014), and the resulting increased nociceptive sensitivity (termed hyperalgesia) is hypothesized to contribute to an increased motivational drive to drink (Egli et al., 2012). In addition to the direct effects of alcohol, abstinence from heavy drinking can also increase affective pain sensitivity (termed hyperkatifeia) as part of a larger motivational withdrawal syndrome (Edwards et al., 2012; Koob, 2021) that may also promote the use of alcohol for affective/emotional pain management. Indeed, self-reports of alcohol use specifically for pain are common. Problem drinkers of both sexes report more severe pain symptoms compared to non-drinkers, and also a higher incidence of using alcohol to manage their pain (Brennan et al., 2005). Interestingly, use of alcohol to manage pain symptoms presaged a worsening of alcohol drinking-related comorbidities, including diabetes (Brennan et al., 2005).

Acute pain represents an adaptive sensory process vital to protecting our bodies from damage. In contrast, chronic or unrelieved pain represents a profound negative emotional experience that can have a powerful influence on brain reinforcement mechanisms, possibly facilitating the transition to AUD in vulnerable individuals (Zale et al., 2021). As support for this conceptualization, there are strong associations among alcohol consumption, chronic pain, and pain-related disability (Witkiewitz et al., 2015a,b; Yeung et al., 2020). Evidence suggests that chronic pain may be predictive of future at-risk alcohol use. In one prospective epidemiological study, self-reported pain interference (or how pain disrupts daily life activities) was predictive of AUD development (McDermott et al., 2018). Additional preclinical research suggests that the relationships between individual levels of alcohol drinking and resultant chronic pain relief may change over time (Adrienne McGinn et al., 2020), although the neurobiological basis of these links is currently unknown.

In addition to chronic pain, important bi-directional relationships exist between at-risk alcohol drinking and other negative affective conditions (e.g., stress, anxiety, depression) that represent additional risks for psychiatric co-morbidities. Importantly, people with chronic pain and high negative affect report higher pain severity and pain interference compared to people with only one of the two disorders (Arnow et al., 2006). Such symptoms may precede or emerge within the development of more severe forms of AUD (Gilpin et al., 2015). For example, the development of negative affective states comprises a portion of the constellation of symptoms of alcohol withdrawal, and as such, likely contribute to negative reinforcement processes that drive continued or escalated drinking over time (Tolomeo et al., 2021). Another intense line of preclinical and clinical investigation concerns factors that precede and may presage the development of at-risk drinking. These range from neurological conditions including traumatic brain injury (Phillips et al., 2014; Mayeux et al., 2015; Adams et al., 2020; Schindler et al., 2021) to pre-existing psychiatric disorders such as post-traumatic stress disorder (Whitaker et al., 2014; Langdon et al., 2016; Livingston et al., 2021).

#### Confounding Factors Impacting Alcohol-Associated Risk for Cardiometabolic Syndrome and Cognitive Deficits

Several biological factors may contribute to alcohol-mediated tissue injury and risk for CMS, including the pattern and type of alcohol use, sex, existing underlying comorbid conditions, and alcohol-induced alterations in target organ milieu (i.e., dysregulation of the extracellular matrix). Both acute and chronic alcohol consumption decrease total brain glucose uptake and the rate of glucose utilization (Volkow et al., 2013), and this may be associated with decreased neuronal activity. These alcohol-associated alterations in brain glucose metabolism may have significant implications for increased risk of cognitive dysfunction, particularly in PLWH (Hammoud et al., 2018; Ge et al., 2021). Binge drinking increases the risk of CMS, type 2 diabetes, stroke, and coronary artery disease compared to continuous alcohol consumption (Lindtner et al., 2013; Hong et al., 2015), and more frequent moderate drinking is associated with more favorable outcomes than occasional or weekly drinking. Moreover, occasional heavy drinking rather than regular heavy drinking is associated with central obesity and hyperglycemia (Wakabayashi, 2014). Increased drinking frequency was associated with increased triglycerides, hyperglycemia, blood pressure, and abdominal obesity only among men (Schroder et al., 2007), and hyperglycemia and hypertension in women (Lee, 2012).

The type of alcohol consumed, and the rate of alcohol metabolism may have a differential impact on CMS risk. Wine that is rich in polyphenols is most associated with decreased CMS incidence (Beulens et al., 2012; Rasouli et al., 2013), and beer in larger doses increases the odds ratio of having a higher waist-hip ratio together with elevated blood pressure and triglycerides (Koloverou et al., 2015; Vieira et al., 2016). Polyphenols found in alcohol, especially resveratrol, are anti-inflammatory and increase eNOS activity, which not only allows for vasodilation but also increases HDL-c transport. Polyphenols also decrease accumulation of 3-nitotyrosine, and PARylated (poly ADP-ribosylated) proteins associated with diabetes (Drel and Sybirna, 2010). Alcohol metabolism differs between sexes. Females have higher percentage of body fat, lower water content, and lower gastric ADH activity than males and these sex-specific differences result in higher blood alcohol concentrations in women than in men given consumption of a similar amount of alcohol (Cowan et al., 1996).

Emerging evidence suggests that Western diets commonly consumed in the U.S. worsen both neurocognitive (Martin and Davidson, 2014; Davidson et al., 2019) and pain symptomatology (Boyd et al., 2021). Thus, the neurobiological interaction of excessive alcohol drinking, cognition, and pain in the context of WD consumption represents a critical area of research and public health interest.

Although alcohol-mediated alterations in extracellular matrix (ECM) remodeling, characterized by an imbalance of extracellular matrisome protein synthesis and degradation leading to overt fibrosis has been well described in alcohol-related liver injury (Poole and Arteel, 2016; Dolin and Arteel, 2020); more recently, dysregulation of matrisome proteins has been reported in adipose, cardiac, SKM, brain, and lung (Sueblinvong et al., 2014; Steiner et al., 2015b; Mouton et al., 2016; Ninh et al., 2019). This altered ECM phenotype has functional consequences that can lead to or exacerbate tissue dysfunction and increase risk for CMS. The major proteins that make up the ECM include collagens, fibronectin, laminin, elastin, and proteoglycans and the continuous remodeling of the matrisome relies on the expression and activity of metalloproteases and their tissue inhibitors (Poole and Arteel, 2016). Studies using both *in vivo* and *in vitro* preclinical models, and postmortem samples of people with AUD have shown that alcohol dysregulates matrisome proteins including laminins, collagen, PAI-1, and tissue plasminogen activator (tPA) (Trindade et al., 2016; Rubio-Araiz et al., 2017; Go et al., 2020). In parallel, alcohol increases AGEs (Vasdev et al., 2006) and the irreversible cross-links of collagens and elastin are unorganized and have dysfunctional ECM fiber distribution (Zieman et al., 2005). AGEs also quench NO and generate peroxynitrite contributing to endothelial dysfunction. In addition, alcohol-mediated oxidative stress induces transforming growth factor beta-1 (TGFβ1) (Sueblinvong et al., 2014), which in turn activates Smad signaling impacting target genes involved in maintaining ECM homeostasis such as PAI-1, urokinase plasminogen activator (uPA), and collagen (Seth et al., 2010). Alcohol dysregulates ECM remodeling producing a profibrotic milieu in SKM (Dodd et al., 2014; Simon et al., 2015) and adipose tissue (Ford et al., 2018) and these alterations in the matrisome are associated with decreased SKM metabolic function and regenerative capacity (LeCapitaine et al., 2011; Simon et al., 2014; Duplanty et al., 2018; Ford et al., 2018) in SIV-infection. Thus, alcohol-induced dysregulation of the ECM contributes to tissue injury and risk of CMS. The ECM plays an important role in brain development, maturation of neural circuits, and adult neuroplasticity, suggesting that processes that affect composition or turnover of brain ECM could impair brain function and contribute to development of neuropsychiatric or neurodegenerative disease (Lubbers et al., 2014; Senkov et al., 2014). Studies show that alcohol affects the regulation of brain ECM through various mechanisms throughout the lifespan. Because of the important role the ECM plays in synaptic processes affected by alcohol exposure, brain ECM remodeling may be an important contributor to the pathophysiology of AUD (Lasek, 2016) and is an area worthy of further investigation.

Taken together, data from clinical and preclinical studies strongly suggest that neuropathological comorbidities in PLWH result from a constellation of pathophysiological mechanisms including metabolic dyshomeostasis, neuroinflammation (Perkins et al., 2019; Delery and Edwards, 2020), and loss of neurotrophic support (Somkuwar et al., 2016; Maxi et al., 2019; Silva-Pena et al., 2019). Comorbid at-risk alcohol use exacerbates these neuropathological processes (Zahr, 2018) and a greater understanding of these mechanisms will lead to new and more effective pharmacological and behavioral strategies for treating AUD (Ray et al., 2021).

## Conclusion

The multisystemic pathophysiological effects of at-risk alcohol use alter underlying cellular and organ homeostasis predisposing the host for increased risk for comorbidities. These alterations are particularly salient in vulnerable populations such as those with chronic diseases, diminishing physiological reserve and increasing vulnerability to tissue injury resulting from direct and indirect effects of alcohol. In PLWH, at-risk alcohol use exacerbates cardiometabolic and neurocognitive pathologies that together with chronic use of ART lead to development of geriatric comorbidities manifested in frailty. Overall, these data strongly support the association of AUD with accelerated biological aging and enhanced risk for comorbidities. Interventions aimed at diminishing at-risk alcohol use are urgently needed in this vulnerable population. Greater understanding of the underlying mechanisms and their specific contribution to comorbidity risk is likely to identify therapeutic targets to ameliorate tissue injury and protect organ systems from the combined impact of HIV infection, chronic ART, and alcohol-mediated tissue injury.

## Author Contributions

LS, SE, and PM conceived and drafted the review, edited, and approved the final version. All authors contributed to the article and approved the submitted version.

## Conflict of Interest

The authors declare that the research was conducted in the absence of any commercial or financial relationships that could be construed as a potential conflict of interest.

## Publisher’s Note

All claims expressed in this article are solely those of the authors and do not necessarily represent those of their affiliated organizations, or those of the publisher, the editors and the reviewers. Any product that may be evaluated in this article, or claim that may be made by its manufacturer, is not guaranteed or endorsed by the publisher.
